# C. A. H. Gee

**Published:** 1938

**Authors:** 


### Obituary

### C. A. H. GEE, M.B., Ch.B.

The death of Dr. C. A. H. Gee, on 7th April, at the age of sixty-one, is a loss to Bristol that will be very keenly felt. He was an old student at the Bristol General Hospital, and after holding a resident appointment there, he commenced practice at Evercreech, Somerset. The war found him a very busy and active practitioner, but the claims of service in the field he found irresistible, and as soon as he could arrange for his work to be carried on, he joined the R.A.M.C. and served in France as a R.M.O. After the Armistice he resumed his practice at Evercreech. Gee had always been interested in skin work, and in 1923 he sold his practice, went to Portbury to live, and came on the B.G.H. staff as assistant to Dr. Kenneth Wills. In 1927 he was appointed Physician in charge of the skin department at the Children's Hospital, and held that appointment at his death. He built up a considerable reputation as a dermatologist, and his kindness and skill will long be remembered amongst a large circle of patients and practitioners. Dr. Gee married Miss M. Wren, a nurse at the Bristol General Hospital, to whom we tender our sincere sympathy. He leaves no family.

